# Identification of Noise Levels for Skill Training Activities, Equipment, Machines and Power Tools at TVET Institutes in Malaysia

**DOI:** 10.3390/ijerph192315783

**Published:** 2022-11-27

**Authors:** Khairul Azhar Abdul Rahim, Jegalakshimi Jewaratnam, Che Rosmani Che Hassan

**Affiliations:** Department of Chemical Engineering, Faculty of Engineering, University of Malaya, Kuala Lumpur 50603, Malaysia

**Keywords:** excessive noise, Technical and Vocational Education Trainings (TVET), noise exposure monitoring

## Abstract

The use of various machines, equipment and power tools at TVET Institute causes the institute’s environment to be exposed to noise hazards that are similar to the industry. However, not much data has been published regarding noise exposure at TVET institutes. This study was carried out to document the noise exposure of work activities training in public TVET institutes in Malaysia that implement skill training programs in metal fabrication, furniture manufacturing and automotive maintenance. The identification of excessive noise, task-based noise exposure monitoring and source measurement was conducted. The noise contribution from each work activity to the daily A-weighted noise exposure level and sound pressure level emitted by machines and equipment was documented. The findings of this study recorded 20 activities with task-based noise contribution to the daily A-weighted noise exposure level between 75.3 dB and 95 dB. Based on the findings, the training environment at the TVET institutes has a risk of operating with excessive noise. The documented data can be used in planning the implementation of suitable noise control measures in TVET institutes.

## 1. Introduction

Noise hazard is one of the most reported hazards in the workplace [1,2,3,4,5]. The result of exposure to excessive noise will cause workers to develop Noise-Induced Hearing Loss (NIHL) disease [6]. To date, NIHL is the most reported occupational disease every year in Malaysia [7,8,9]. Since it is synonymous with the employment sector, control and prevention are more focused in this sector. In fact, noise hazard studies and investigations by previous researchers were mostly done in the context of the employment sector [3]. It was found that most occupational sectors expose workers to noise levels above regulatory limits [3,10,11,12,13,14,15,16,17]. This situation has caused stricter controls to be enforced to regulate noise exposure [18,19]. Furthermore, NIHL disease is also chronic in nature, as it exposes workers to noise for a long period of time [20,21]. Based on these characteristics, noise exposure, starting at a young age, can cause significant early NIHL effects. Therefore, intervention should start earlier, especially in adolescents.

Many studies have reported a pattern of NIHL cases among adolescents [22,23,24]. This happens due to the emergence of several factors, such as noise exposure from recreational activities and lifestyle [25,26]. In addition, adolescents are also exposed to noise in educational settings [27,28,29]. Since they spend a significant amount of time in educational institutes, uncontrolled noise exposure will cause them to develop premature NIHL symptoms. With a low level of awareness and poor hearing protection practices, the risk of ear damage among them can be high [30,31,32,33]. One of the educational institutions in Malaysia that is particularly at risk of exposing students to noise is the Technical and Vocational Education and Training (TVET) Institution [34,35].

TVET is dubbed a ‘game changer’, as it is a way for the Malaysian government to tackle one of the country’s primary issues, i.e., the lack of skilled manpower [36,37]. Graduates of these institutes are targeted to become a skilled workforce in various industrial sectors. In order to ensure that TVET graduates have the competencies required by future employers, the TVET Training environment has been designed to match the actual workplace environment [38]. However, this comes with a price. The educational setting that is designed to imitate a real-life workplace environment inadvertently causes students to be exposed to the same hazards as in the industry [39,40]. The use of noisy heavy duty hand tools and machines has put students at risk of NIHL [34,35,41,42]. Apart from that, students are also exposed to noise in their classes [43,44,45,46]. This situation is not only hazardous for the students, but it is also a risk for the trainers [47]. Among the TVET programs that are at risk of exposing students and trainers to noise are furniture manufacturing, automotive maintenance and metal fabrication [34,48,49,50]. These areas are indeed known to have noisy environments in the real industry [14].

The furniture manufacturing industry is an industry that has various hazards [51]. Other than exposure to wood dust that affects the respiratory system, noisy environments also endanger workers. Among carpentry works that are carried out are cutting, edging, sanding and shaping wood to produce furniture. The use of machines and hand tools such as various types of saws cause the industrial environment to be excessively noisy [52]. Meanwhile, the automotive industry is considered as one of the main industries that offers various job opportunities. Having to operate with various machines, this industry also exposes workers to loud noises [53,54]. Apart from engine maintenance, work activities in this industry also include chassis repair, painting and metal fabrication work. Next, the metal manufacturing industry is also identified to expose workers to noise hazards [10,13,55]. Activities such as metal cutting, grinding, shearing and welding are known to produce high noise levels [56,57]. In 2020, an occupational noise-related hearing disorder (ONRHD) report on 7941 cases was confirmed by the DOSH [7]. This figure represents 87% of the total confirm occupational disease and continues to be the most reported occupational disease in Malaysia. The manufacturing sector is reported to have the most confirmed NIHL cases [58]. Evidently, in these industries, various types of machines, equipment and power tools are used by workers [59]. These devices are indeed known to operate in a noise range that exceeds the healthy limit, which puts workers in danger.

This study was conducted to investigate the noise levels for skill-training activities, equipment, machines and power tools at TVET institutes in Malaysia. Three TVET institutes that offer programs in furniture manufacturing technology, automotive technology and metal fabrication technology were selected to participate in this study. Through this study, the identification of an excessive noise risk assessment was carried out in all three institutes. Noise exposure monitoring and source measurement were conducted to record and documenting noise levels from work activities, equipment, machines and power tools used in the institutes. The data from this study are expected to provide an overview of the noise exposure situation at TVET institutes. These data can then be used in developing a suitable intervention plan to be executed in TVET institutes.

## 2. Materials and Methods

### 2.1. Data Collection

Three public TVET institutes were selected for the purpose of data collection. These institutes were selected because they offer TVET programs that are known to be noisy in nature and have a potential to expose students and trainers to excessive noise [14]. For the purpose of this study, the names of these institutes are identified by A, B and C. Institute A is located in the state of Sarawak and offers metal fabrication program. Institute B is located in the state of Selangor and offers a furniture manufacturing program, while institute C is located in the state of Negeri Sembilan, and the program offered is automotive maintenance. Permission to conduct research and data collection was obtained from the director general of these institutions through written and email applications. This study was carried out from 4 February 2019 to 21 January 2022. To contain the widespread COVID-19 virus, the Malaysian government implemented a series of Movement Control Orders starting from 18 March 2020 [60]. During the lockdown, the education sector was especially affected, as students were prohibited to go to schools. However, the data collection process during this period was collected when these three institutes were having ongoing teaching and learning activities onsite via a hybrid approach for their full-time program students. This study also received ethical approval from the University of Malaya Research Ethics Committee (UMREC) (Ref. No: UM.TNC2/UMREC_1073). A participant information sheet containing the purpose and procedure of the study was explained and distributed to the study participants before the study began. In addition, consent forms were distributed and collected again before the study was carried out.

### 2.2. Identification of Excessive Noise

The identification of excessive noise was conducted before noise exposure monitoring. It was carried out based on the nature of the activities and workshop area. The identification was carried out by the institute’s safety and health coordinator through the Identification of Excessive Noise survey form. The survey form was adopted from the latest industrial code of practice published by the Department of Occupational Safety and Health (DOSH) Malaysia [61]. The form consisted of 10 questions, and respondents were asked to answer them in the form of ‘yes’ or ‘no’. Should there be at least one ‘yes’ answer, the nature of the activities and workshop area could be considered to have a risk of excessive noise exposure; thus, a noise risk assessment should be carried out. The research form needed to be verified by the respondent and the administrator of the institutes involved.

### 2.3. Noise Exposure Monitoring

In order to study the level of noise emitted in the institutes, noise exposure monitoring was carried out. It was conducted by recording the noise level by area, machine and work activity. For work activities, the monitoring was conducted using a Noise Dosimeter 3M Edge eg-4 (3M-Quest Technology, Oconomowoc, WI, USA). The measurements were conducted based on the International Standard of Acoustic Determination of Occupational Noise Exposure Engineering Method (ISO 9612) [62]. The dosimeter was chosen, because it meets the requirements of IEC 61252. The dosimeters measured the A-weighted sound pressure level in decibels (dB) and was fixed at the criterion level of 85 dB, 3 dB exchange rate, and 80 dB threshold. The dosimeter was calibrated before and after each measurement. The microphone was mounted on the top of the shoulder and placed at a distance of 0.1 m from the ear canal on the most-exposed side and was approximately 0.04 m above the shoulder. The microphone was fastened in such a way that mechanical influence or covering by clothing did not lead to false results of the measured student’s body. A task-based measurement strategy was used to determine the noise exposure for each task that was probably repeatable. Based on the interview with the trainers, tasks (*m*) were identified and the duration (${\bar{T}}_{m}$) for each task was determined based on actual duration in an academic schedule. Three measurements were recorded per task with a measurement period of 5 min for each work cycle. The A-weighted equivalent continuous sound pressure level for each task was calculated using Equation (1):

(1) $$L_{p\mathrm{Aeq}Tm}=10\;lg\left(\frac{1}{I}\sum_{i=1}^{I}10^{0.1\times L_{p\mathrm{Aeq}Tmi}}\right)dB$$

where *L_p_*_Aeq*Tm*_ is the A-weighted equivalent continuous sound pressure level during a task of duration *T_m_*, *i* is the number of task sample *m* and *I* is the total number of task samples *m*. The noise contribution from each task to the daily A-weighted noise exposure level was then determine using Equation (2):

(2) $$L_{\mathrm{EX}8hm}=L_{p\mathrm{Aeq}Tm}+10lg\left(\frac{{\bar{T}}_{m}}{T_{o}}\right)dB$$

where *T_m_* is the duration of task *m* and *T_o_* is the reference duration, *T_o_* = 8 h. Since this study only focus on the individual task noise exposure level, the daily A-weighted noise exposure level, *L_EX8h_*, was not calculated.

The maximum A-weighted sound pressure level, *L_max_*, emitted by machines and equipment use in the institutes’ workshops were measured using a Sound Level Meter (SLM) CPS SM150 kit (CPS, Miramar, FL, USA). The SLM, along with its accessories, met the standards of IEC 61672-1:2002, Class 2 instrumentation and was calibrated before and after each measurement. The *L_max_* recorded designated the maximum sound level that occurred during the observation period. Therefore, it could be used to identify the source that emits a high level of noise. Furthermore, over a period of time, the maximum sound level was higher than average value; hence, it was more suitable to be used in determining the safest control measure. The measurements were conducted based on the standard and guidelines from the Department of Occupational Safety and Health (DOSH) Malaysia [61]. The SLM was fixed at a high range (65~130 dB), time-weighting ‘S’ and ‘A’ frequency weighting. The noise level was measured using the spot sampling method, and the microphone of the sound level meter was pointed towards the direction of the noise source at 1 meter from the ground and 1 meter away from noise source. The maximum A-weighted sound pressure level, *L_max_*, was calculated based on 3 readings recorded per noise source with time intervals of 5 min each. Noise readings for equipment, power tools and vehicle were recorded in minimal background noise where no other work activities were carried out during the measurements.

### 2.4. Statistical Analysis

Statistical analysis for the noise source measurements was performed using IBM SPSS Statistics for Windows version 25 (IBM Corporation, Armonk, NY, USA). A 95% confidence interval (CI) was obtained for each source measurement. A paired-sample *t*-test was conducted to determine the significant difference between the A-weighted maximum sound pressure level, *L_max_* recorded and the sound levels reported by the published database [59]. 

### 2.5. Intervention

The outcome of this study was presented to the administration of the institutes, along with a recommendation on the control measures. Additionally, information regarding hearing conservation awareness was distributed to the students and trainers of the institutes. An hour of educational programs regarding NIHL prevention was conducted for them as well.

## 3. Results

### 3.1. Identification of Excessive Noise

The result of the Identification of Excessive Noise survey is shown in Table 1. Institute A, which is located in the city of Kota Samarahan, Sarawak, and implements a metal fabrication technology program, substantiated the potential of noise exposure on six items. Three items did not show the potential for noise exposure, while the tenth item is stated to be not applicable, since the noise risk assessment has never been carried out. Institute B, which is located in Kuala Langat, Selangor, and offers a furniture manufacturing technology program, verified the potential of noise exposure for the seven items asked. However, two items did not show the potential for noise exposure. Similar to institute A, institute B also stated not applicable to item 10. Institute C located in Chembong, Negeri Sembilan, showed the potential for noise exposure for six items that were asked, while no potential for noise exposure was detected for the remaining four items. The guidelines published by the DOSH stated that at least one verification to any of the identification item indicates the possibility of excessive noise [61]. Therefore, these institutes have potential for excessive noise exposure, and based on the requirements of the law, they need to carry out further noise risk assessments.

### 3.2. Work Activities Noise Exposure Monitoring

Table 2 shows the results of work activities noise exposure measurements for institute A, where task-based measurements were conducted on five activities. The calculated A-weighted equivalent continuous sound pressure level shows the work activities in the metal fabrication workshop emitted noise levels ranging from *L*_*p*Aeq*T*_ 89.4 dB to 103.7 dB. Cutting metal using a metal cut-off saw recorded the highest noise level, while the lowest noise was recorded from SMAW welding work activity. The highest noise contribution to the daily A-weighted noise exposure level was also recorded by cutting metal using a metal cut-off saw with *L_EX,8hm_* 94.7 dB, while the lowest noise contribution was recorded by oxy fuel metal plate cutting with *L_EX,8hm_* 80.5 dB.

Table 3 shows the results of work activities noise exposure measurements for institute A, where task-based measurements were conducted on nine activities. The calculated A-weighted equivalent continuous sound pressure level shows the work activities in the furniture manufacturing workshop emitted noise levels ranging from *L*_*p*Aeq*T*_ 84.2 dB to 91.1 dB. Spray paint furniture recorded the highest noise level, while the lowest noise was recorded from wood edging work activity. The highest noise contribution to the daily A-weighted noise exposure level was also recorded by spray paint furniture with *L_EX,8hm_* 85.1 dB. The lowest noise contribution was recorded by wood mortiser with *L_EX,8hm_* 76.2 dB.

Table 4 shows the results of work activities noise exposure measurements for institute C, where task-based measurements were conducted on six activities. The calculated A-weighted equivalent continuous sound pressure level shows the work activities in the automotive maintenance workshop emitted noise levels ranging from *L*_*p*Aeq*T*_ 84.3 dB to 101.0 dB. Car body painting in a spray booth recorded the highest noise level, while the lowest noise was recorded from the work activity of rollcage installation. Similarly, the highest and lowest task-based noise contributions to the daily A-weighted noise exposure level were also recorded by same work activities with *L_EX,8hm_* 95.0 dB and 75.3 dB, respectively. 

### 3.3. Equipment, Power Tools and Vehicle Noise Exposure

The means of the A-weighted maximum sound pressure level, *L_max_* for equipment, power tools and vehicles used in these three TVET programs are as shown in Table 5. The highest reading was obtained from the metal cut-off saw with *L_max_* of 104.1 (2.30) dB (95% CI, 98.4–109.8 dB). This was followed by the spray booth for an automotive maintenance workshop with *L_max_* of 101.4 (2.72) dB (95% CI, 94.6–108.2 dB). Sixteen items recorded readings above 90 dB. The remaining 31 items recorded readings between 85.1 dB and 89.9 dB, with the lowest reading recorded from the training vehicle simulator (Sedan) (95% CI, 84.4–92.6 dB). There was a statistically significant difference in *L_max_* recorded (m = 90.4, SD = 4.5) and the A-weighted sound levels reported by a published database (m = 93.4, SD = 9.25); *t*(46) = −2.74, *p* = 0.009. Overall, the maximum sound pressure level readings for the equipment, power tools and vehicle were statistically significantly lower than the Malaysia permissible A-weighted maximum sound pressure level of 115 dB (*t*(46) = −24.623, *p* = 0.000).

## 4. Discussion

The operational settings at all study locations were found to have risk of emitting excessive noise. Based on the requirements outlined in the industrial code of practice, if the respondent gives at least one ‘yes’ answer to any questions on the Identification of Excessive Noise assessment, then the area can be considered to have the potential for excessive noise exposure [61]. Therefore, further assessments need to be conducted. In this study, institutes A, B and C each gave ‘Yes’ answers to six, seven and six items respectively. All three institutes stated that there are communication difficulties in the training area due to the noisy environment. The finding shows that TVET institutes poses additional noise exposure risk aside from the classroom noise [45]. The risk of noise exposure is also high considering that these institutes also use equipment that is known to emit high noise levels. In addition, there is also a source of impulsive noise in these institutes. However, complaints regarding hearing problems are at a minimum. This may be because low in awareness among students and trainers on the issues of noise exposure and hearing loss. The risk of noise exposure was never known by the management of the institute since these institutes never carried out a noise risk assessment or audiometric testing.

Metal fabrication is known to be one of the noisiest industries [58]. In this study, A-weighted equivalent continuous sound pressure level has been recorded from five work activities. The sound level recorded is almost similar with the level found in the industrial environment [55]. Cutting of material using metal cut-off saw is the noisiest work activity. Based on observation, the machine is used on a daily basis where students use it to cut their training material. Therefore, this exposure can cause chronic effects of NIHL on them in the future. In addition, sources of impulsive noise were also detected in the workshop environment, especially from shearing work activities. The level of noise produced depends on the thickness and type of metal [63]. Grinding activity is also one of the activities that are often carried out in the field of metal fabrication, and it is one of the activities that known to produce high noise level [57]. During the training time, the entire workshop area is used by students (15–25 students per group) where they perform various work activities training according to their assigned group. This causes high background noise in the workshop area. Furthermore, the indoor setting cause echoes in the workshop area. The calculated daily A-weighted noise exposure level contributed by each training activities, *L_EX,8h,m_* shows students are exposed to noise level above 80 dB for each training period. This situation is feared to contribute to the daily A-weighted noise exposure level exceeding the allowed noise exposure limit.

For the furniture manufacturing program, A-weighted equivalent continuous sound pressure level has been recorded from nine work activities. Furniture manufacturing related industry is known to expose workers to various hazards [51]. The prolong use of machines and hand tools causes students and trainers to be in a noisy environment, hence, exposing them to adverse effects of noise exposure. Similar to the metal fabrication program, the noise emitted depends on the type and thickness of the material [52,64,65]. Among the types of wood used for training at this institute are Nyatoh, Rubberwood, Meranti, Balau, Pinewood and Merbau. The furniture manufacturing workshop also operates daily with various training activities and use of various machines running simultaneously. This causes high background noise during training. Spray paint furniture contribute the highest task’s daily A-weighted noise exposure level. This was followed by sharpening tool points and part assembly, with both recorded *L_EX,8h,m_* exceeding 80 dB. Similar to the metal fabrication program, furniture manufacturing has a potential to expose students and trainer to daily A-weighted noise exposure level exceeding the allowed noise exposure limit if the amount of noise from other activities is added.

In the automotive workshop, noise exposure monitoring has been conducted for the training module of engine repair work, structural repair, final finishing and heavy vehicle maintenance. Most of the work activities use similar hand tools as in the metal fabrication program. Therefore, noise level recorded is almost the same. The A-weighted equivalent continuous sound pressure level recorded for each work activity showed that the training workshop emitted high noise level similar as has been reported in the industry [54,66]. The highest noise is recorded from the spray painting in the spray booth. The source of the noise in the booth is from the air compressor and exhaust fan [67]. In addition, the automotive maintenance workshop also emitted background noise from the sound of various training vehicles. High noise level was recorded when heavy vehicles are turned on.

The result from the statistical analysis suggests the recorded A-weighted maximum sound pressure level, *L_Max_* from equipment, power tools and training vehicles are slightly lower from the A-weighted sound levels, *L_A_* reported by published database [59]. The highest noise was recorded from the metal cut-off saw which is known to produce high noise level during the cutting of iron rods [59,68]. Manufacture information regarding noise was only found in some equipment, power tools and training vehicles. However, this is not known to the user. This may be due to the lack of awareness and education programs regarding noise exposure among them. Based on the recorded *L_Max_* data, TVET institutes have the potential to have similar noise hazards as industrial environments. 

Based on observations, the three institutes have not implemented the Hearing Conservation Program (HCP). Noise control is more focused on the provision of Hearing Protection Devices (HPD). However, the use of HPD by students and trainers were found to be not consistent. The available HPD also does not follow the appropriate attenuation rate. Furthermore, students and trainers admitted not knowing about noise reduction rating (NRR). Therefore, the effectiveness of protection should be questioned. Additional engineering control was not implemented by the institute to control noise. Administrative control is implemented by scheduling student training; however, it is based on curriculum structure and not specifically to control noise exposure. Although there are noise warning signs, a hearing protection zone has yet to be designated officially. Previous noise exposure studies at the educational institution found excessive noise level in the classroom [9a–c]. By considering these findings, students and trainers at the TVET institutes faced with a higher risk of noise exposure compared to nontechnical education institutes, since their theoretical learning in the classroom and also during practical training in the workshop exposed them to excessive noise levels.

### Recommendation

Based on the results of the excessive noise identification study, TVET institutes need to conduct complete noise exposure monitoring as outlined in the Occupational Safety and Health (Noise exposure) Regulation 2019. This study only focuses on documenting the noise contribution from work activities to the daily A-weighted noise exposure level and the noise level that emitted by machines, equipment and hand tools used in three TVET programs. It is recommended that a complete personal noise exposure monitoring is conducted to measure the daily personal noise exposure levels. Furthermore, audiometric tests can also be carried out to find out the level of hearing of students and trainers at TVET institutes. It is recommended for the similar study to be conducted in other TVET programs.

## 5. Conclusions

The findings of this study show that all three TVET institutes involved have a risk of excessive noise exposure. The noise monitoring conducted has successfully document the level of noise contribution from each work activity of the TVET training modules to the daily A-weighted noise exposure level. These data can be used by trainers to design an appropriate and safe training schedule. The value of noise contribution from each work activity can be used to identify the combination of work activities that are safe and will not exposed students and staff to the daily noise exposure level above the permissible exposure limit. At the same time, these data can be used in planning the implementation of suitable noise control measures. The procurement of HPD can also be guided by the noise value that is documented. This study has also managed to record the value of the A-weighted maximum sound pressure level ***L_Max_*** from equipment, power tools and training vehicles used in the TVET institutes involved. Based on these data, ‘buy quite policy’ can be formulate. As an intervention, TVET institutes are recommended to implement the Hearing Conservation Program (HCP). HCP needs to be implemented since the TVET institutes are also subject to the Occupational Safety and Health Act 1994. As an institute that produces skilled workers, TVET institute needs to play a role in producing workers who have awareness and have good hearing protection practices in the future.

## Figures and Tables

**Table 1:** Results of noise hazard identification.

No	Excessive Noise Identification Questions	Institute/Location/Program		
		A	B	C
		Kota Samarahan, Sarawak	Kuala Langat, Selangor	Chembong, Negeri Sembilan
		Metal Fabrication	Furniture Manufacturing	Automotive Maintenance
1	Is a raised voice needed to communicate with someone about one meter away?	Yes	Yes	Yes
2	Do your employees notice a reduction in hearing over the course of the day?	No	Yes	No
3	Are your employees using noisy powered tools or machinery?	Yes	Yes	Yes
4	Are there noises due to impacts or explosive sources?	Yes	Yes	Yes
5	Are personal hearing protectors (PHP) used for some work?	Yes	Yes	Yes
6	Do your workers complain that there is too much noise or that they cannot clearly hear instructions or warning signals?	Yes	Yes	No
7	Do your workers experience ringing in the ears or sound heard differently in each ear?	No	No	No
8	Has any employee start experiencing difficulties in hearing after working here?	No	No	No
9	Does any equipment have manufacturer’s information (including labels) indicating noise levels greater than any of the following: (a) peak sound pressure level of 140 dB(C)? (b) sound pressure level of 82 dB(A)?	Yes	Yes	Yes
10	Is the latest noise risk assessment indicates exposure to Noise Exposure Limit?	N/A	N/A	Yes

**Table 2:** A-weighted equivalent continuous sound pressure level and noise contribution from task *m* to the daily A-weighted noise exposure level results for work activities in the metal fabrication workshop.

No	Task (*m*)	*L*_*p*Aeq*Tm*_ (dB)	Duration (*h*)	*L_EX,8h,m_* (dB)
1	Grinding and Cutting	95.7	2	89.7
2	TIG and MIG Welding	89.6	2	83.6
3	SMAW Welding	89.4	2	83.4
4	Cutting-Plate/Sheet (Oxy)	89.5	1	80.5
5	Cutting-Bar (Cut-Off Saw)	103.7	1	94.7

**Table 3:** A-weighted equivalent continuous sound pressure level and noise contribution from task *m* to the daily A-weighted noise exposure level results for work activities in the furniture manufacturing workshop.

No	Task (*m*)	*L*_*p*Aeq*Tm*_ (dB)	Duration (*h*)	*L_EX,8h,m_* (dB)
1	Wood cutting	85.3	1	76.3
2	Wood edging	84.2	2	78.2
3	Part assembles	87.4	2	81.4
4	wood mortiser	85.2	1	76.2
5	wood rounding	85.4	1	76.4
6	wood curving	84.5	2	78.5
7	sharpening tool points	90.4	1	81.4
8	spray paint furniture	91.1	2	85.1
9	Surface abrading	86.2	1	77.2

**Table 4:** A-weighted equivalent continuous sound pressure level and noise contribution from task *m* to the daily A-weighted noise exposure level results for work activities in the automotive maintenance workshop.

No	Task (*m*)	*L*_*p*Aeq*Tm*_ (dB)	Duration (*h*)	*L_EX,8h,m_* (dB)
1	Installing exhaust components	87.9	2	81.9
2	Engine Maintenance	87.3	2	78.3
3	Rollcage Installation	84.3	2	75.3
4	Installation of vehicle body parts	85.1	2	79.1
5	Car Body Painting (Spray booth)	101.0	2	95.0
6	Metal Work—Cutting & Grinding	91.00	1	82.0

**Table 5:** Equipment, power tools and vehicle A-weighted maximum sound pressure level, *L_max_*.

No	Equipment/Power Tools/Vehicle	Workshops	*L_max_* (dB) (SD)	95% CI (dB)	A-Weighted Sound Levels Reference, *L_A_*
1	Thicknesser planer	Furniture Making	99.7 (1.35)	96.4, 103.0	107.0
2	Jointer planer	Furniture Making	96.6 (1.51)	92.8, 100.4	106.0
3	Lathe machine	Furniture Making	88.7 (1.29)	85.5, 91.9	89.0
4	Band saw	Furniture Making	89.9 (1.56)	85.9, 93.8	90.0
5	Portable jig saw	Furniture Making	89.3 (1.12)	86.5, 92.1	97.0
6	Scroll saw	Furniture Making	88.9 (1.16)	86.0, 91.7	98.0
7	Mitre saw	Furniture Making	97.9 (2.36)	92.0, 103.8	103.0
8	Sliding table saw	Furniture Making	90.1 (1.75)	85.8, 94.5	92.0
9	Metal cut-off saw	Metal Fabrication	104.1 (2.30)	98.4, 109.8	113.0
10		Automotive Maintenance	99.5 (0.65)	97.9, 101.2	113.0
11	Angle grinder	Metal Fabrication	90.8 (2.75)	84.0, 97.6	84.0
12		Automotive Maintenance	92.0 (3.97)	82.2, 101.9	84.0
13	Bench grinder	Furniture Making	89.4 (0.7)	87.7, 91.1	84.0
14		Metal Fabrication	93.1 (0.45)	92.0, 94.3	84.0
15		Automotive Maintenance	89.8 (0.85)	87.7, 91.9	84.0
16	Portable router	Furniture Making	89.1 (1.05)	86.5, 91.7	93.0
17	Bench drilling machine	Furniture Making	88.2 (1.00)	85.7, 90.7	87.0
18	Cordless drill	Furniture Making	89.4 (0.76)	87.5, 91.3	98.0
19		Metal Fabrication	91.2 (1.46)	87.5, 94.8	98.0
20		Automotive Maintenance	89.8 (1.32)	86.5, 93.0	98.0
21	Spray booth	Furniture Making	86.8 (1.80)	82.4, 91.3	105.0
22	Spray booth	Automotive Maintenance	101.4 (2.72)	94.6, 108.2	105.0
23	Air compressor	Furniture Making	88.7 (0.56)	87.3, 90.1	94.0
24		Metal Fabrication	89.6 (1.59)	85.7, 93.6	94.0
25		Automotive Maintenance	88.9 (0.79)	86.9, 90.9	94.0
26	Orbital sander	Furniture Making	90.2 (2.02)	85.2, 95.2	94.0
27		Automotive Maintenance	79.9 (4.96)	67.6, 92.3	94.0
28	Belt sander	Furniture Making	88.1 (2.10)	82.9, 93.3	95.0
29	Sanding machine	Furniture Making	89.2 (2.09)	84.0, 94.4	97.0
30	Surface grinding machine	Metal Fabrication	89.3 (1.08)	86.6, 92.0	102.0
31	Shear machine	Metal Fabrication	95.3 (0.85)	93.2, 97.4	93.0
32	TIG welding Machine	Metal Fabrication	87.4 (1.93)	82.6, 92.2	74.0
33		Automotive Maintenance	85.8 (1.59)	81.9, 89.7	74.0
34	SMAW welding Machine	Metal Fabrication	85.4 (0.76)	83.4, 87.3	79.0
35		Automotive Maintenance	85.8 (1.05)	83.2, 88.4	79.0
36	Fume extractor	Metal Fabrication	92.9 (0.56)	92.9, 91.5	105.0
37	Oxy acetylene torch	Metal Fabrication	87.3 (0.78)	85.4, 89.3	89.0
38	Plasma arc cutting machine	Metal Fabrication	89.6 (3.00)	82.1, 97.0	100.0
39	Engine (diesel) on stand	Automotive Maintenance	91.2 (1.35)	87.9, 94.6	80.0
40	Air impact wrench	Automotive Maintenance	93.6 (1.31)	90.4, 96.9	103.0
41	Training Vehicle (Sedan)	Automotive Maintenance	85.1 (0.85)	83.0, 87.2	90.0
42	Forklift	Metal Fabrication	88.5 (1.66)	84.4, 92.6	89.0
43		Automotive Maintenance	86.5 (0.76)	84.6, 88.4	89.0
44	Bulldozer (Heavy Vehicle)	Automotive Maintenance	86.9 (1.59)	82.9, 90.8	98.0
45	Truck (Heavy Vehicle)	Automotive Maintenance	88.7 (1.06)	86.1, 91.4	90.0
46	Tractor (Heavy Vehicle)	Automotive Maintenance	89.6 (0.74)	87.8, 91.5	93.0
47	Backhoe (Heavy Vehicle)	Automotive Maintenance	88.3 (0.66)	86.7, 89.9	87.0

## Data Availability

Our data are available upon request from the corresponding author.
